# Robot-assisted versus laparoscopic total gastrectomy: a western high-volume tertiary referral center experience

**DOI:** 10.1007/s11701-026-03451-0

**Published:** 2026-05-20

**Authors:** Jonas Sanberg, Bo J. Noordman, Mats Lindblad, Fredrik Klevebro, Ioannis Rouvelas

**Affiliations:** 1 Department of Upper Abdominal Surgery, Center for Digestive Diseases, Karolinska University Hospital, Huddinge, Stockholm, 141 86 Sweden; 2 Department of Surgery, Odense University Hospital, Odense, Odense C, DK-5000 Denmark; 3 Division of Surgery and Oncology, Department of Clinical Science, Intervention and Technology (CLINTEC), Karolinska Institutet, Huddinge, Stockholm, C177, 141 86 Sweden

**Keywords:** Gastrectomy, Robotic surgical procedures, Laparoscopy, Postoperative complications, Anastomotic leak

## Abstract

Robot-assisted gastrectomy has been increasingly adopted as a standard of care, but comparative evidence in Western cohorts remains limited, particularly for total gastrectomy, where esophagojejunostomy and D2 lymphadenectomy are technically demanding. The aim of this study was to compare short-term outcomes after robot-assisted and laparoscopic total gastrectomy at a European high-volume tertiary referral center. This retrospective single-center cohort study included all patients undergoing minimally invasive total gastrectomy (laparoscopic or robot-assisted) for gastric cancer or prophylaxis in CDH1-mutated patients at Karolinska University Hospital (Stockholm, Sweden) from 1 January 2015 to 31 August 2025. The primary outcome was any postoperative complication within 30 days. Secondary outcomes included anastomotic leak, intraoperative blood loss, severe complications (Clavien–Dindo grade ≥ 3), length of stay (LOS), lymph node yield, and 90-day mortality. In total, 156 patients were included (robot-assisted, n=58; laparoscopic, n=98). Any complication occurred in 37.9% after robot-assisted surgery and 68.4% after laparoscopic surgery (P<0.001). Anastomotic leak occurred in 10.3% and 6.1%, respectively (P=0.364). Median blood loss was lower with robot-assisted surgery (50 vs 125 mL, P<0.001). Median LOS, lymph node yield, and 90-day mortality were comparable between groups. In multivariable analyses, robot-assisted surgery was associated with significantly lower odds of any complication (adjusted OR 0.22, 95% CI 0.08–0.56; *P* = 0.002). There were no statistically significant differences between groups regarding anastomotic leak (adjusted OR 0.73, 95% CI 0.10–5.54; *P* = 0.761) or severe complications (adjusted OR 0.95, 95% CI 0.32–2.87; *P* = 0.933). In this Western single-center cohort from a high-volume tertiary referral center, robot-assisted total gastrectomy demonstrated reduced postoperative morbidity to laparoscopic surgery and was associated with reduced intraoperative blood loss.

**Supplementary Information:**

The online version contains supplementary material available at 10.1007/s11701-026-03451-0.

## Introduction

Minimally invasive gastrectomy is established in curative gastric cancer surgery, aiming to reduce perioperative morbidity compared to open gastrectomy, without compromising oncologic adequacy in appropriately selected patients treated in high-volume experienced centers [1, 2]. Total gastrectomy remains technically demanding because suprapancreatic D2 lymphadenectomy and intracorporeal esophagojejunostomy are performed in constrained anatomy, with outcomes influenced by case volume, patient habitus, and perioperative pathways—factors that differ substantially between East Asian and Western practice [1, 3–5].

Robot-assisted platforms may mitigate some technical constraints of minimally invasive total gastrectomy through articulated instruments, tremor filtration, stable three-dimensional visualization, and improved surgeon ergonomics [6]. However, comparative evidence has been dominated by East Asian series, and external validity to Western cohorts characterized by a higher BMI distribution, greater comorbidity burden, and broader use of perioperative chemotherapy, remains uncertain [7–9].

Recent high-volume meta-analytic evidence (2022–2023) comparing robotic versus laparoscopic gastrectomy suggests lower blood loss and fewer severe complications with robotic surgery, with broadly similar overall morbidity and oncologic surrogates, albeit with longer operative time and persistent risks of residual confounding due to predominantly nonrandomized source studies [10–12]. Total-gastrectomy–specific comparative data remain limited but continue to suggest potential reductions in postoperative complications and modest differences in lymph node yield in selected settings [13]. Accordingly, Western center data with contemporary perioperative care and clinically relevant endpoints (including standardized complication grading) are needed [7, 14].

The present study aimed to compare early postoperative outcomes after laparoscopic versus robot-assisted total gastrectomy in a Western high-volume tertiary referral center, focusing on clinically meaningful morbidity and incorporating multivariable adjustment for key patient, tumor, and treatment-related confounders [14].

## Methods

### Study design and setting

This retrospective single-center cohort study was conducted at the Department of Upper Abdominal Surgery, Center for Digestive Diseases, Karolinska University Hospital,, Stockholm, Sweden, a high-volume tertiary referral center for upper gastrointestinal cancer surgery. The study period spanned from 1 January 2015 to 31 August 2025. Minimally invasive gastrectomy was introduced at our institution in 2012, as previously described [15], and robot-assisted gastrectomy was implemented in 2021.

## Participants

All patients undergoing minimally invasive total gastrectomy for gastric adenocarcinoma or as prophylaxis due to CDH1 mutation during the study period were included in the study. Procedures were performed either laparoscopically or with robotic assistance. The exposure of interest was the surgical approach (laparoscopic versus robot-assisted). Patients converted to open surgery were analyzed according to the intention-to-treat principle based on the initially intended minimally invasive approach.

## Surgical technique

The unit has access to the DaVinci^®^ Xi robotic system (Intuitive Surgical, Sunnyvale, CA, USA); laparoscopic procedures were performed using a three-dimensional (3D) high-definition camera system. In robot-assisted total gastrectomy, four robotic trocars and one assistant port were placed. Gastric mobilization and lymphadenectomy were performed in a standardized fashion and were comparable between the two approaches.

All anastomoses were completed intracorporeally using a side-to-side linear stapling technique, regardless of the surgical approach. The esophagojejunostomy was fashioned using an EndoWrist^®^ 30 mm blue load cartridge (Intuitive Surgical) in robotic cases, and an Endo-GIA^®^ Tri-Staple™ 30 mm brown load cartridge (Medtronic, Minneapolis, MN, USA) in laparoscopic cases. The enteroenterostomy was constructed using a SureForm^®^ 60 mm blue load cartridge (Intuitive Surgical) in robotic cases, and an Endo-GIA^®^ Tri-Staple™ 60 mm brown load cartridge in laparoscopic cases.

In all cases, the stapler entry site was closed with full-thickness interrupted sutures using a 4 − 0 absorbable barbed suture (V-Loc^®^, Medtronic). Perioperative management was standardized in accordance with the institutional early recovery program.

All procedures were performed by four senior surgeons experienced in minimally invasive upper gastrointestinal surgery, using a standardized technique across both approaches.

## Outcomes

The primary outcome was any postoperative complication within 30 days of surgery. This was defined as any deviation from the expected postoperative course, including both minor and major events requiring medical intervention, and was prospectively recorded in the institutional database at discharge and at postoperative follow-up visits. Complications were graded according to the Clavien–Dindo classification [14]. Severe complications were defined as Clavien–Dindo grade ≥ 3.

Secondary outcomes included anastomotic leak, intraoperative blood loss, operative time, length of hospital stay (LOS), number of resected lymph nodes, conversion rate, readmission within 30 days, mortality (90-day and in-hospital), and initiation of adjuvant therapy after neoadjuvant treatment.

Anastomotic leak was defined as an endoscopically and/or radiologically confirmed anastomotic dehiscence with contrast extravasation. Routine postoperative imaging was not performed; imaging or endoscopic assessment was undertaken only in patients with clinical suspicion of a leak.

## Covariates

Covariates included age, sex, Body Mass Index (BMI, kg/m^2^), ECOG performance status, ASA class, Charlson comorbidity index (CCI) [16], tumor location, histological type (adenocarcinoma versus CDH1-associated prophylactic resection), (y)cTNM8 stage [17], year of surgery, and preoperative oncological therapy. For preoperative cTNM-staging, ycTNM was used for patients, who had received preoperative chemotherapy.

### Statistical analysis

Categorical variables were compared using the Chi-square test. Continuous variables were analyzed using Student’s t-test or log-rank test where appropriate according to normal or non-normally distributed data. Fisher’s exact test was used to compare 30-day mortality rates due to low event counts. P values of 0.05 or less were considered significant.

Multivariable logistic regression was used to calculate adjusted odds ratios (ORs) to estimate relative risk for [1] any complication [2], anastomotic leak [3], severe complications (Clavien–Dindo ≥ 3), and [4] non-initiation of adjuvant therapy after preoperative treatment. The adjusted model included predefined clinically relevant covariates selected based on existing literature and biological plausibility, including age (continuous), sex, BMI category, ECOG performance status, ASA class, (y)cTNM8 stage, tumor location, histological type, and neoadjuvant treatment. Mortality was not modeled due to the limited number of events.

To illustrate the possible learning curves for both robot-assisted and laparoscopic total gastrectomy, anastomotic leak rates, lymph node yield, duration of surgery, and blood loss were plotted across consecutive cases in 5-case intervals for both groups.

## Ethics

Ethical approval was obtained from the Regional Ethics Review Board in Stockholm, reference numbers 2018/970–31/1, 2020–01459, 2021–02810, 2023–02802-02, 2024–03122-02, and 2025-03744-02.

## Results

### Study population

During the study period, 162 patients underwent minimally invasive total gastrectomy; 156 were eligible for inclusion in this study, comprising 58 in the robot-assisted group and 98 in the laparoscopic group. Baseline characteristics according to surgical approach are presented in Table 1. Median age was similar in the robot-assisted and laparoscopic groups (69 vs. 67 years, *P* = 0.476). The robot-assisted group had a higher proportion of female patients (60.3% vs. 37.7%; *P* = 0.008) and a more favorable ECOG distribution (ECOG 0: 74.1% vs. 54.1%; *P* = 0.009), while ASA class 3 was more frequent in the robot-assisted group (51.7% vs. 30.6%; *P* = 0.035). Tumor location differed between groups, with gastroesophageal junction tumors (Siewert II) observed only in the robot-assisted group (13.8% vs. 0%; *P* = 0.001). Clinical stage distributions also differed between groups, with more favorable tumor staging in the robot-assisted group (*P* = 0.033).

**Table 1:** Baseline characteristics of patients undergoing laparoscopic versus robot-assisted total gastrectomy

	Robot-assisted TG	Laparoscopic TG	*P*-value
	*N* = 58	*N* = 98	
**Age (years**,** median**,** range)**	69 (25–86)	67 (27–86)	0.476
**Sex (n**,** %)**			0.008*
- Male	23 (39.7)	61 (62.2)	
- Female	35 (60.3)	37 (37.7)	
**Body Mass Index**,** kg/m**^**2**^ **(n**,** %)**			0.469
- < 18,5	2 (3.5)	6 (6.1)	
- 18,5–24,9	31 (53.5)	42 (42.9)	
- 25–29,9	14 (24.1)	33 (33.7)	
- 30+	11 (19.0)	17 (17.4)	
**ECOG Performance Status (n**,** %)**			0.009*
- 0	43 (74.1)	53 (54.1)	
- 1	15 (25.9)	35 (35.7)	
- 2	0 (0.0)	10 (10.2)	
**ASA-score (n**,** %)**			0.035*
- 1	6 (10.3)	23 (23.5)	
- 2	22 (37.9)	44 (44.9)	
- 3	30 (51.7)	30 (30.6)	
- 4	0 (0.0)	1 (1.0)	
**Charlson Comorbidity Index (median**,** range)**	0 (0–4)	0 (0–7)	0.417
**Indication for gastrectomy (n**,** %)**			0.027
- Adenocarcinoma	48 (82.8)	92 (93.9)	
- CDH1 mutation	10 (17.2)	6 (6.1)	
**Tumor location (n**,** %)**			0.001*
- GEJ / Proximal	26 (44.8)	30 (30.6)	
- Corpus	14 (24.1)	48 (49.0)	
- Antrum	8 (13.8)	10 (10.2)	
- Pylorus	0 (0.0)	1 (1.0)	
- Other/no tumor	10 (17.2)	9 (9.2)	
**Preoperative oncological therapy (n**,** %)**	30 (51.7)	57 (58.2)	0.434
**(y)cTNM8-stage (n**,** %)**			0.033*
- 0	10 (17.2)	6 (6.1)	
- I	13 (22.5)	12 (12.2)	
- II	25 (43.1)	60 (61.2)	
- III	10 (17.2)	18 (18.4)	
- IV	0 (0.0)	2 (2.0)	

### Perioperative outcomes

Median operative time was similar between the robot-assisted and laparoscopic groups (322.5 vs. 315.5 min, *P* = 0.870). Median estimated blood loss was lower after robot-assisted surgery (50 vs. 125 mL, *P* < 0.001). Conversion to open surgery occurred in 8/58 (13.8%) robot-assisted cases and 23/98 (23.5%) laparoscopic cases (*P* = 0.154). Perioperative outcomes are summarized in Table 2.

**Table 2:** Perioperative and 90-day postoperative outcomes after laparoscopic versus robot-assisted total gastrectomy

	Robot-assisted TG	Laparoscopic TG	*P*-value
	*N* = 58	*N* = 98	
**Duration of surgery (minutes**,** median**,** range)**	322.5 (210–651)	315.5 (160–720)	0.870
**Blood loss ml (median**,** range)**	50 (10-2000)	125 (0-2000)	< 0.001*
**Conversion (n**,** %)**	8 (13.8)	23 (23.5)	0.154
**Any complication (n**,** %)**	22 (37.9)	67 (68.4)	< 0.001*
**Anastomotic leak (n**,** %)**	6 (10.3)	6 (6.1)	0.364
**Highest Clavien-Dindo-score (n**,** %)**			< 0.001*
- 0	36 (62.1)	31 (31.6)	
- I-II	6 (10.3)	47 (47.9)	
- III-IV	14 (24.1)	19 (19.4)	
- V	2 (3.5)	1 (1.0)	
**Length of stay (days**,** median**,** range)**	6 (2–43)	7 (4–65)	0.086
**Number of resected lymph nodes (median**,** range)**	39 (19–121)	42 (0-143)	0.123
**Radicality (n**,** %)**			0.463
- R0	55 (96.5)	88 (90.7)	
- R1	2 (3.5)	7 (7,2)	
- R2	0 (0.0)	2 (2.1)	
**(y)pTNM8 stage (n**,** %)**			0.006*
- 0	9 (15.5)	6 (6.1)	
- I	19 (32.8)	17 (17.3)	
- II	10 (17.2)	32 (32.7)	
- III	19 (32.8)	39 (39.8)	
- IV	1 (1.7)	4 (4.1)	
**Readmission within 30 days**	8 (13.8)	15 (15.3)	1.000
**90-day mortality (n**,**%)**	2 (3.5)	1 (1.0)	0.286
**In-hospital mortality (n**,**%)**	1 (1.7)	0 (0.0)	0.192
**Initiation of adjuvant oncological therapy in patients who received neoadjuvant treatment**	23 (76.7)	47 (82.5)	0.517

Learning curve analyses for anastomotic leak, lymph node yield, operative time, and estimated blood loss are shown in the Supplementary Figure S1, illustrating trends across sequential operative cases.

### Primary outcome

Any complication within 30 days occurred in 22/58 (37.9%) after robot-assisted surgery and 67/98 (68.4%) after laparoscopic surgery (*P* < 0.001). The distribution of Clavien–Dindo grades differed significantly between groups (*P* < 0.001). (Table 2)

### Secondary outcomes

Severe complications (Clavien–Dindo ≥ 3) occurred in 16/58 (27.6%) robot-assisted cases and 20/98 (20.4%) laparoscopic cases (*P* = 0.311). Anastomotic leak occurred in 6/58 (10.3%) robot-assisted cases and 6/98 (6.1%) laparoscopic cases (*P* = 0.364). Median LOS was 6 days after robot-assisted surgery and 7 days after laparoscopic surgery (*P* = 0.090). Lymph node yield was similar between groups (median 39 vs. 42, *P* = 0.123), with one patient in the laparoscopic group having 0 lymph nodes reported due to incomplete histopathological assessment following early postoperative death. R0 resection was achieved in 96.5% of robot-assisted cases and 90.7% laparoscopic cases (*P* = 0.460).

Readmission within 30 days occurred in 13.8% and 15.3% of patients in the robot-assisted and laparoscopic groups. respectively (*P* = 1.00). Ninety-day mortality occurred in two patients in the robot-assisted group and one patients in the laparoscopic group (3.5% vs. 1.1%; *P* = 0.286). In-hospital mortality was 1.7% after robot-assisted surgery and 0% after laparoscopic surgery (*P* = 0.192). Adjuvant oncological therapy in patients who had received neoadjuvant treatment was initiated in 76.7% of robot-assisted patients and 82.5% of laparoscopic patients (*P* = 0.517). (Table 2)

### Multivariable analyses

In adjusted logistic regression, robot-assisted surgery was associated with a lower odds of any postoperative complications (adjusted OR 0.22, 95% CI 0.08–0.56; *P* = 0.002). There were no statistically significant differences between groups regarding anastomotic leak (adjusted OR 0.73, 95% CI 0.10–5.54; *P* = 0.761) or severe complications (adjusted OR 0.95, 95% CI 0.32–2.87; *P* = 0.933). Robot-assisted surgery showed a tendency towards lower odds of non-initiation of adjuvant therapy after neoadjuvant treatment, although the difference was not statistically significant (adjusted OR 0.24, 95% CI 0.04–1.28; *P* = 0.095). Adjusted associations between surgical approach and postoperative outcomes are shown in Table 3.

**Table 3:** Adjusted associations between surgical approach and postoperative outcomes

Outcome	Adjusted OR (Robot vs. Laparoscopic)	95% CI	*P* value
Any complication	0.22	0.08–0.56	0.002
Anastomotic leak	0.73	0.10–5.54	0.761
Severe complications (Clavien–Dindo ≥ 3)	0.95	0.32–2.87	0.933
Non-initiation of adjuvant therapy after neoadjuvant treatment	0.24	0.04–1.28	0.095

Multivariable logistic regression models were adjusted for age, sex, BMI category, ECOG performance status, ASA class, tumor location, histological type, clinical TNM stage, year of surgery and neoadjuvant therapy

## Discussion

In this Western single-center cohort of 156 minimally invasive total gastrectomies, robot-assisted surgery demonstrated postoperative outcomes comparable to laparoscopy within 90 days. Overall complication rate was reduced in robot-assisted surgery, and anastomotic leak rate did not differ significantly between approaches. Robot-assisted surgery was associated with less blood loss.

Our findings does not align with the most recent meta-analysis of robot-assisted versus laparoscopic total gastrectomy, which reported no significant difference in overall complications (RR 0.91) and anastomotic leak (RR 0.73), but a lower risk of severe complications (Clavien–Dindo ≥ 3; RR 0.65), reduced blood loss, shorter hospital stay, and slightly higher lymph node yield with robotic surgery [18]. In our cohort, blood loss was also reduced with robot-assisted surgery, whereas lymph node yield and LOS were similar. Notably, we did not observe a reduction in severe complications, which may reflect differences in case mix, surgical technique, learning curve effects, or perioperative pathways in a Western setting, despite a reduction in complications in general.

A key contribution of this study is the provision of Western real-world data focused specifically on total gastrectomy. The existing comparative literature is dominated by East Asian cohorts, and the 2025 meta-analysis included only studies from China, Japan, and Korea. Western patients frequently differ with respect to BMI, comorbidity burden, tumor location, and patterns of multimodal therapy. In our cohort, tumor location differed significantly between groups, with gastroesophageal junction tumors more frequently treated with the robot-assisted approach. Such anatomical complexity may influence the difficulty of reconstruction and the profile of complications, highlighting the importance of accounting for potential confounding when comparing surgical approaches.

The adjusted OR for any complication suggested a clinically relevant reduction with robot-assisted surgery, although the reduction was observed only for mild complications (Clavien-Dindo < 3). This finding should be interpreted cautiously. The distribution of Clavien–Dindo grades differed substantially between groups, with a higher proportion of patients without complications after robot-assisted surgery. However, the proportion of severe complications was not lower, suggesting that the robotic approach may influence minor morbidity more than major adverse events in this setting.

In this study, complication rates after minimally invasive total gastrectomy are higher than those reported in most contemporary series. In the laparoscopic arm of the CLASS02 randomized trial, the overall complication, leak, and grade III–V complication rates were 19.1%, 1.0%, and 7.6%, respectively [19]. In the Japanese JCOG1401 confirmatory study, which included both total and proximal gastrectomy and is therefore only partially comparable, grade 2–4 esophagojejunal leakage was 2.5%, and in-hospital grade 3–4 adverse events were 29% [20].

Among contemporary robotic versus laparoscopic total gastrectomy cohorts, Yang et al. reported 23.8% versus 28.6% overall morbidity, 2.4% versus 1.6% anastomotic leakage, and 5.6% versus 5.6% major complications after matching [21]. Hikage et al. [22] and Shibasaki et al. [23] reported lower robotic morbidity compared with laparoscopy in high-volume Japanese centers, and Nagata et al. found grade ≥ 3 complications of 1% versus 11% after matching [13]. Total-gastrectomy–specific pooled evidence is directionally similar: Chen et al. found no significant difference in overall complications or leak, but fewer major complications with robotic total gastrectomy (OR 0.59, 95% CI 0.38–0.93) [24], whereas Manara et al. reported descriptive rates of approximately 16% overall complications, 2% leak, and 6% severe complications for robotic total gastrectomy versus 18%, 3%, and 6% for lap-assisted total gastrectomy [25].

Overall, the complication rates reported in the present study, particularly in the laparoscopic group, are higher than those observed in most contemporary high-volume series. This is likely explained by differences in complication definitions and reporting practices, as well as potential variation in patient case-mix and perioperative pathways. Importantly, in the present study, all deviations from the expected postoperative course were systematically recorded as complications, including minor and self-limiting events, which may contribute to the higher observed rates.

Anastomotic leak remains a key quality indicator after total gastrectomy. The leak rate in our cohort was within the range reported in the literature. The adjusted model did not show a difference between approaches, consistent with meta-analytic estimates [18]. Adjuvant therapy initiation is a clinically important endpoint integrating postoperative recovery and functional status. We observed a higher proportion commencing adjuvant therapy in the robot-assisted group, and adjusted analyses suggested lower odds of not starting adjuvant therapy, although this was not statistically significant. Although exploratory, this observation may indicate that minimally invasive recovery after robotic surgery could facilitate timely postoperative oncological treatment and warrants further study in larger Western datasets.

This study has limitations inherent to its retrospective design. Selection bias and unmeasured confounding cannot be excluded, and the surgical approach may have been influenced by surgeon preference, resource availability, and temporal changes in practice. After the introduction of robot-assisted gastrectomy, patients continued to undergo either robotic or laparoscopic surgery depending on platform availability and operative scheduling. However, baseline characteristics suggested comparable comorbidity profiles between groups, although differences in clinical stage were observed. Multivariate analyses were therefore performed to adjust for clinically relevant confounders.

The study period spans a decade during which perioperative care, enhanced recovery program implementation, implementation of FLOT, and robotic adoption may have evolved. Additionally, the sample size limits power for uncommon outcomes, including mortality and anastomotic leak, and precluded adjusted modeling for mortality. The potential influence of a learning curve for robotic gastrectomy should also be considered. No significant learning-curve signal was observed in this study, which may reflect the institution’s extensive experience with robot-assisted upper gastrointestinal surgery, including robotic esophagectomy [26]. Importantly, the robotic program was implemented substantially later than the laparoscopic program, yet comparable perioperative outcomes were achieved within a relatively short implementation period.

A cost-effectiveness analysis of laparoscopic versus robot-assisted total gastrectomy was not performed in the present study. Previous meta-analytic data suggest that robot-assisted gastrectomy is associated with higher procedural costs compared with laparoscopy [27]. However, it remains unclear whether potential clinical advantages, such as reduced complication rates or shorter length of stay, may offset these additional costs. Furthermore, variability in institutional resources, surgical volume, and perioperative care pathways may substantially influence the economic balance between the two approaches. As such, the overall cost-effectiveness of robot-assisted gastrectomy remains context-dependent, and further prospective studies integrating clinical outcomes with comprehensive cost analyses are warranted to better define its value.

## Conclusions

In a Western tertiary center, robot-assisted total gastrectomy demonstrated reduced 30-day morbidity to laparoscopic surgery and was also associated with reduced blood loss. Although adjusted analyses demonstrated fewer postoperative complications with the robotic approach, statistical significance was not reached when analyzing serious complications. These findings support the use of robot-assisted total gastrectomy in specialized centers, while highlighting the need for larger Western studies incorporating long-term oncologic outcomes and cost-effectiveness.

## Electronic Supplementary Material

Below is the link to the electronic supplementary material.

Supplementary Material 1Learning curves analyses for robot-assisted total gastrectomy illustrating anastomotic leak rates (A), lymph node yield (B), operative time (C), and blood loss (D). Data are aggregated in sequential groups of five consecutive cases to visualize trends over time during the implementation of the robotic program.

## Data Availability

Data is not publicly available due to Swedish law.
