# Supplementary figures and images for: Robot-assisted versus laparoscopic total gastrectomy: a western high-volume tertiary referral center experience
Source: J Robot Surg. 2026 May 20;20(1):512. doi: 10.1007/s11701-026-03451-0 (PMC13186787; doi:10.1007/s11701-026-03451-0)

**Supplementary Figure S1**

**A**


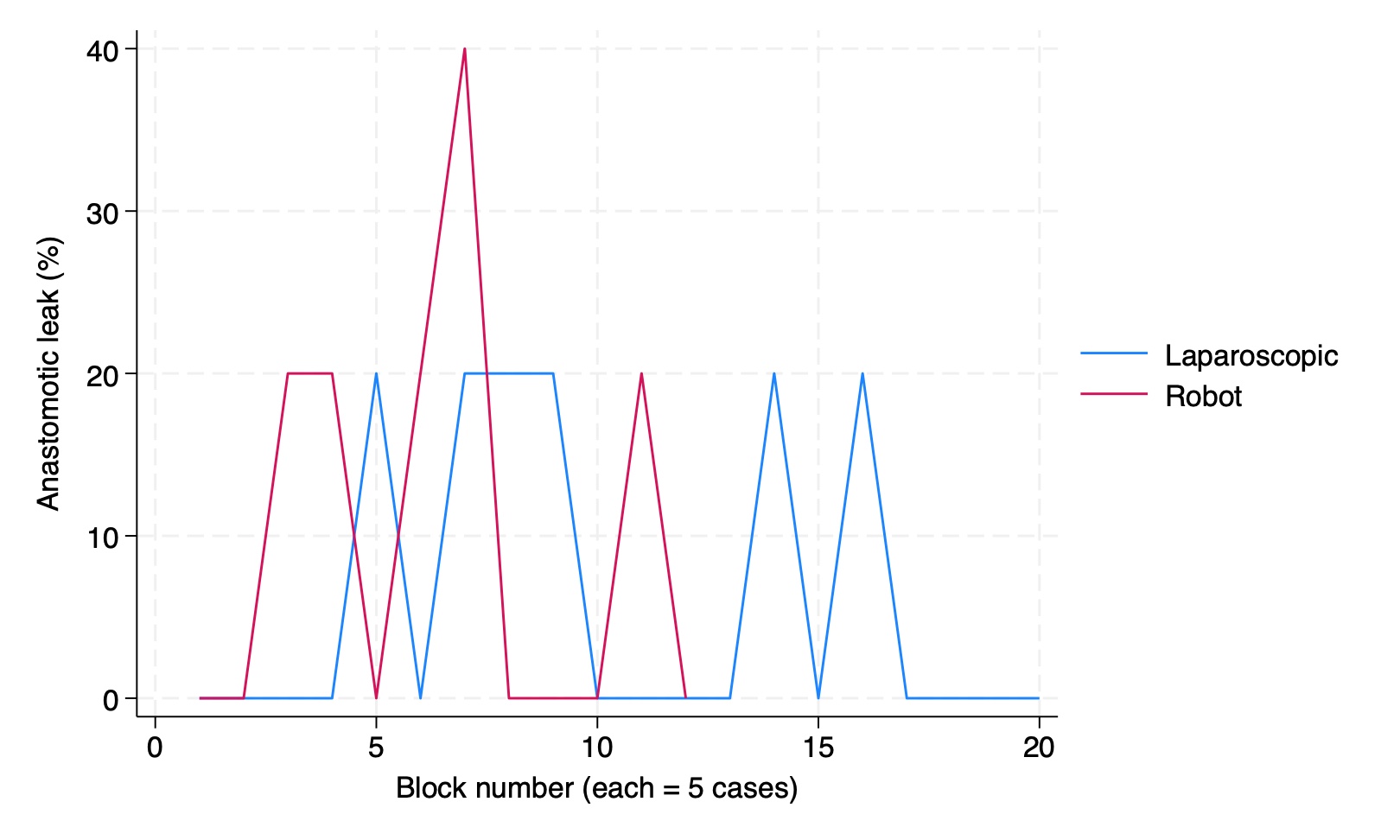
**B**
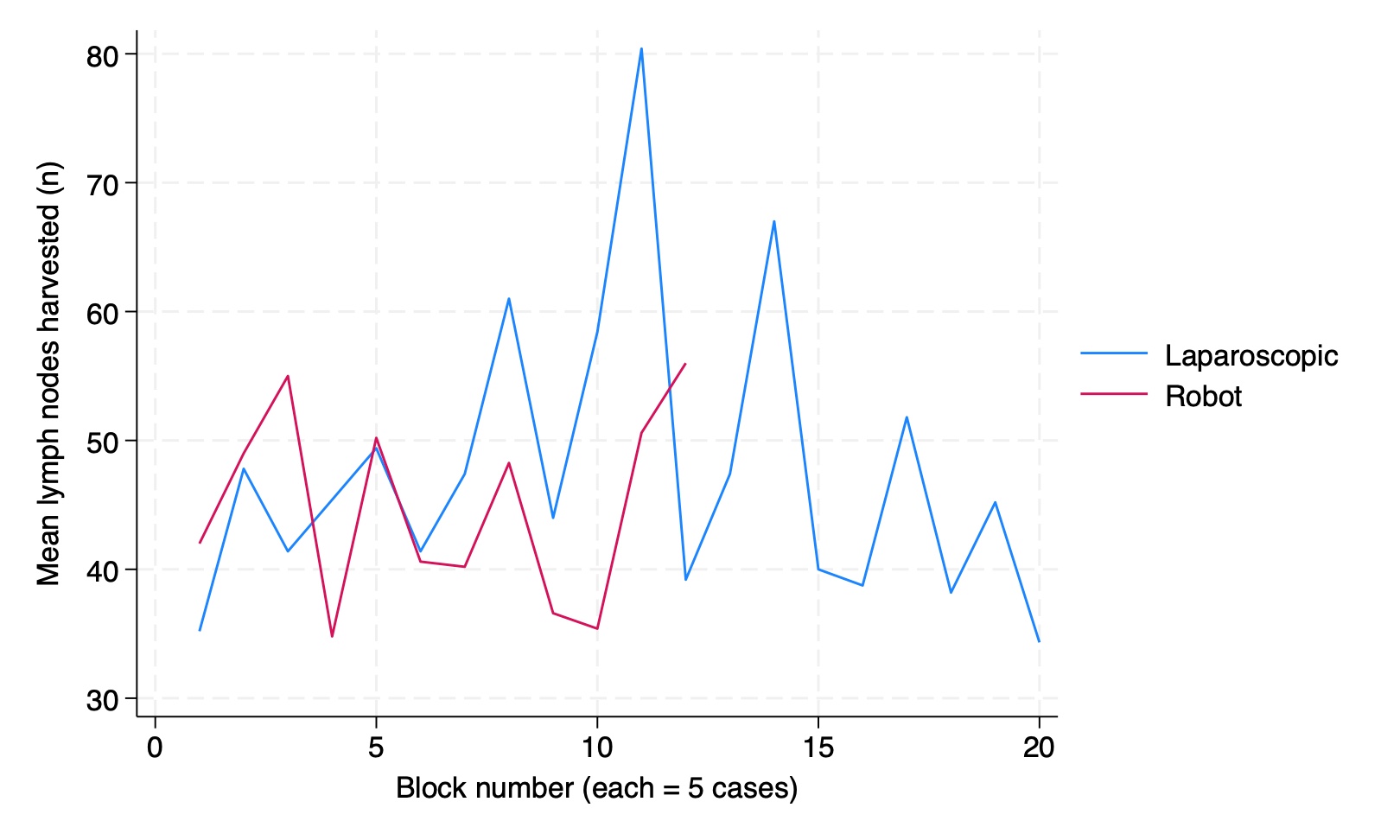


**C**
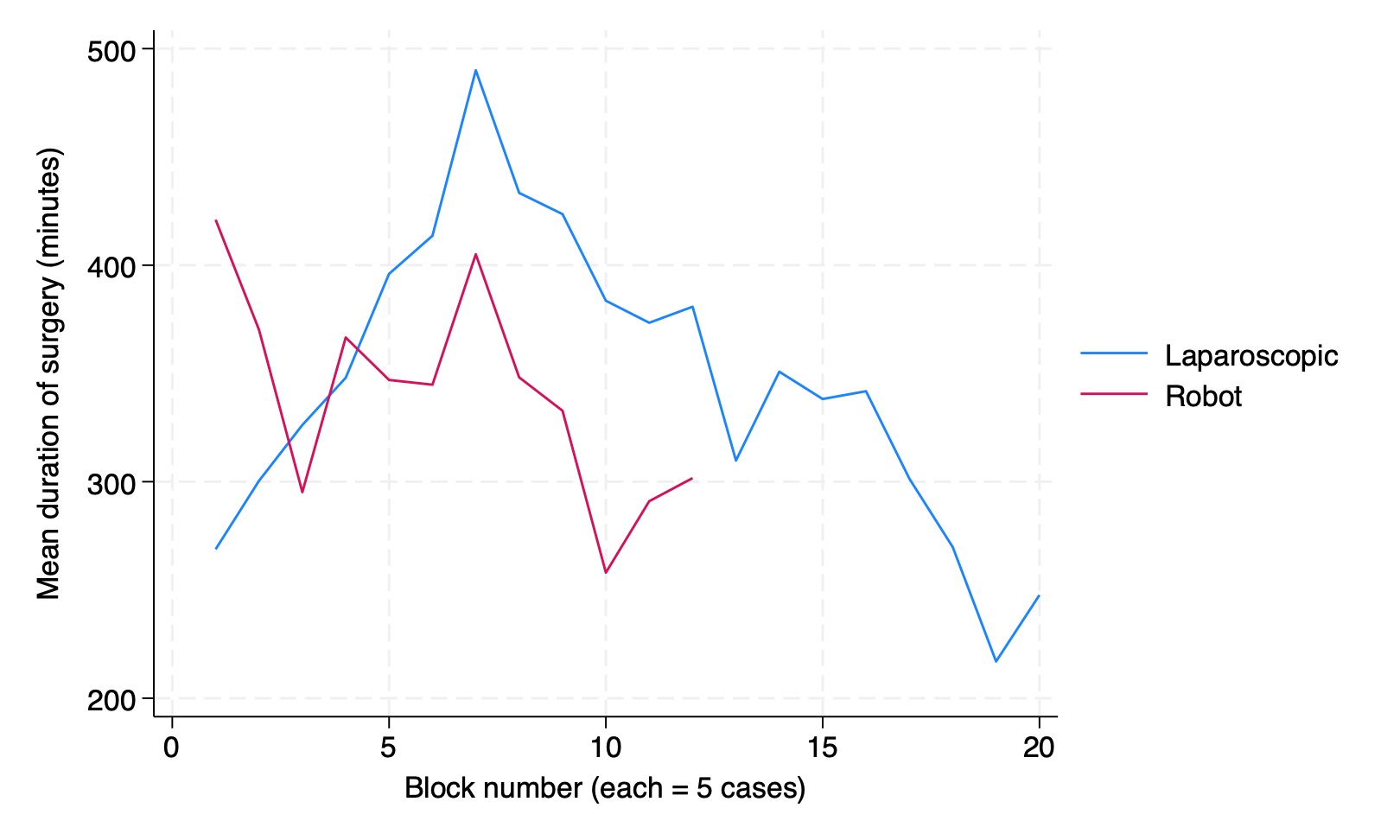
**D**
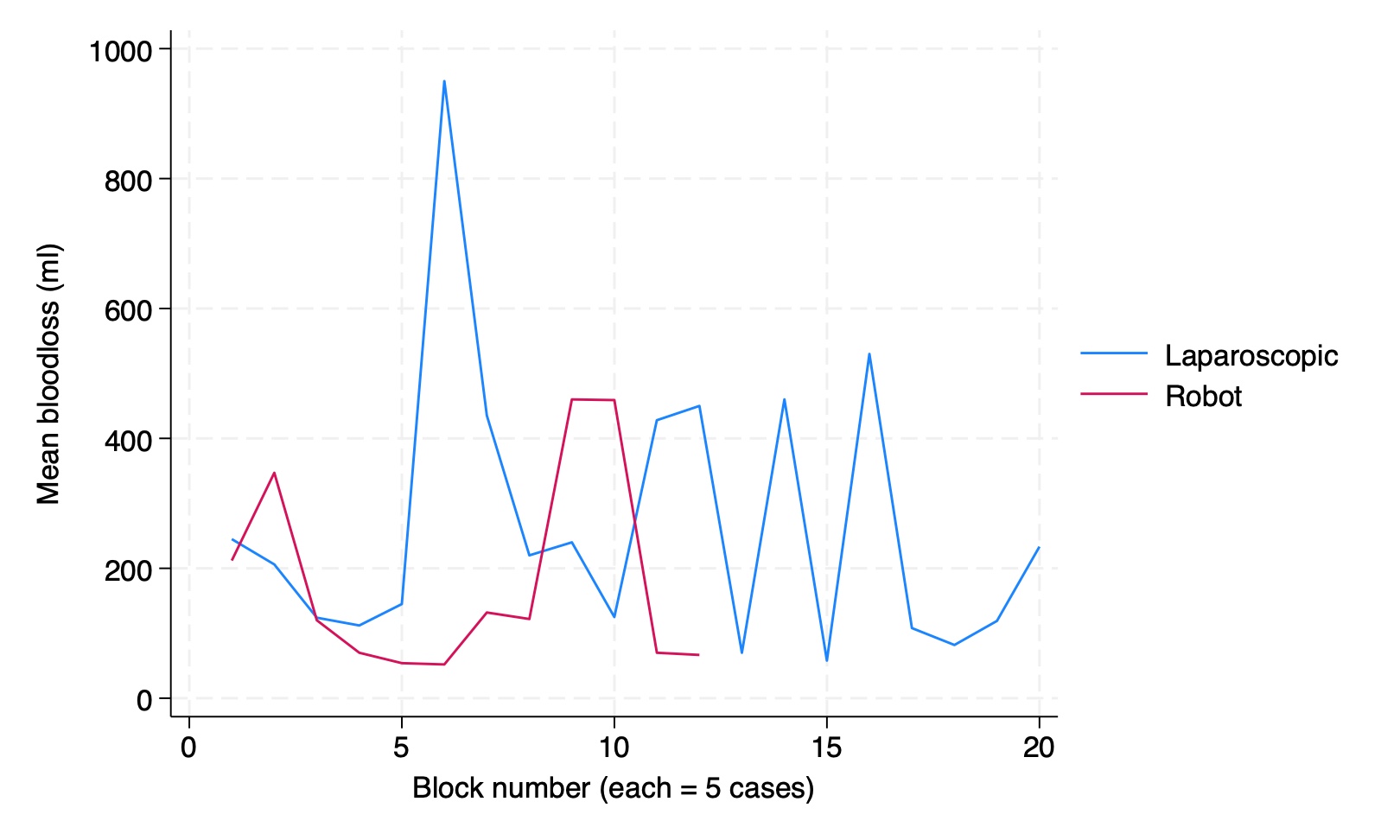

Supplement: Supplementary file 1 — Supplementary Material 1Learning curves analyses for robot-assisted total gastrectomy illustrating anastomotic leak rates (A), lymph node yield (B), operative time (C), and blood loss (D). Data are aggregated in sequential groups of five consecutive cases to visualize trends over time during the implementation of the robotic program. [file 11701_2026_3451_MOESM1_ESM.docx]
